# Autoimmune Thyroid Disease and Sjögren Disease: Organ-Specific Disease Triggered by Systemic Autoimmunity?

**DOI:** 10.3390/medicina61020287

**Published:** 2025-02-07

**Authors:** Atalay Dogru, Fatma Gur Hatip

**Affiliations:** Division of Rheumatology, Department of Internal Medicine, Faculty of Medicine, Süleyman Demirel University, Isparta 32260, Turkey; drfatmagur@gmail.com

**Keywords:** anti-nuclear antibody, autoimmune thyroiditis, salivary gland, Sjögren disease

## Abstract

*Background and Objectives*: Autoimmune thyroid diseases are more prevalent in patients diagnosed with Sjögren disease (SD) than in the general population. SD and autoimmune thyroid diseases are two distinct yet interrelated autoimmune disorders. The objective of this study was to determine the frequency of autoimmune thyroiditis (AT), autoantibody relationships, and clinical features in patients with SD. *Materials and Methods:* The study included 525 patients. A retrospective evaluation was conducted on the demographic data, biochemical and serological tests, and pathological data of the patients. An anti-nuclear antibody (ANA) test was performed using the indirect immunofluorescence (IIF) method using HEp-2 (HEp-2000) cells as substrate. The Schirmer test and minor salivary gland biopsy were conducted on all patients. *Results:* AT was detected in 167 (31.8%) of 525 patients who participated in the study. The anti-nuclear antibody (ANA) test and anti-SS-A positivity rate were higher in the AT group (*p* value < 0.001 and 0.002 respectively). We found that the likelihood of developing AT increased as ANA titres increased. ANA positivity titres were found to be significant at 2+, 3+, and 4+ values (odd ratios 2.41, 3.40, and 4.21, respectively). Additionally, histological examination of salivary gland biopsies revealed a significantly higher prevalence of diffuse lymphocytic infiltration in the AT group. *Conclusions:* AT was present in 31% of patients with SD. The presence of ANA positivity, anti-SS-A positivity, and diffuse lymphocytic infiltration appears to exert an influence on the association between these two diseases.

## 1. Introduction

Sjögren disease (SD) is a systemic autoimmune disease with a complex pathogenesis, primarily affecting the exocrine glands. Patients frequently report persistent oral and ocular dryness resulting from the progressive infiltration of salivary and lacrimal glands by lymphocytes and plasma cells. The vast majority of patients (92% for dry eyes and 94% for dry mouth) experience these symptoms. The ratio of female to male patients is 9:1, with a marked female predominance. The fourth and fifth decades are the most common ages at which the disease starts. Despite being one of the most prevalent autoimmune disorders observed in the general population, a significant proportion of patients remain undiagnosed due to the disease’s insidious progression and the absence of overt symptoms [1,2]. In one-third of patients, systemic findings are present. The most common extraglandular involvement is interstitial lung disease in the lungs. However, the disease may affect many systems, including the skin, joints, muscles, peripheral and central nervous systems, and the haematological system. The pathogenesis of the disease has yet to be fully elucidated; however, it is postulated to be a multifactorial process involving environmental triggers that lead to an aberrant immune response in individuals with a genetic predisposition [3].

Autoimmune thyroid diseases represent the most well-known examples of autoimmune diseases affecting a single organ. The most prevalent thyroid gland diseases are Graves’ disease and chronic lymphocytic thyroiditis (Hashimoto’s disease) [4]. In Graves’ disease, antibody development against the thyrotropin receptor and the subsequent activation of thyroid follicular cells are observed. It is the most common cause of hyperthyroidism. In Hashimoto thyroiditis, there is a diffuse infiltration of lymphocytes into the thyroid parenchyma. Immune activation occurs as a result of MHC class II expression. The process initiated by T cell infiltration results in the destruction of thyroid cells, leading to the development of hypothyroidism. Hashimoto thyroiditis is the most common cause of hypothyroidism, affecting approximately 10% of the global population [5].

There are multiple pathways in the pathogenesis of SD, involving genes in both human leukocyte antigens (HLAs) and non-HLA regions. The HLA locus is the strongest genetic factor for susceptibility. In particular, HLA class II alleles DRB1*03:01, DQA1*05:01, and DQB1*02:01 are important risk factors for the development of SD. It is evident that non-HLA genes, polymorphisms, and epigenetic modifications contribute to the emergence of diseases. Co-occurrence with other autoimmune diseases, such as thyroid diseases with a common genetic background, is observed. It has been demonstrated that thyroid and epithelial cells express the same HLA molecules belonging to class II, with HLA-B8 and HLA-DR3 being of particular significance in this regard [6,7].

It is well documented that autoimmune thyroid diseases are more prevalent in patients diagnosed with SD than in the general population. SD and autoimmune thyroid diseases are two distinct yet interrelated autoimmune disorders. Hashimoto thyroiditis represents the most prevalent autoimmune thyroid disease in SD. The lacrimal gland and salivary gland share functional and structural similarities with the thyroid gland. The cascade of events observed in SD, including lymphocytic infiltration of exocrine glands and glandular damage, is similar in autoimmune thyroid gland diseases. The infiltration of CD4+ T cells and B cell activation are common in both diseases [8,9]. The objective of this study was to investigate the frequency of autoimmune thyroiditis (AT), autoantibody relationships, and clinical features in patients with SD.

## 2. Materials and Methods

The study included 525 patients aged 18 years and over, of Turkish ethnicity, who were followed up in the Rheumatology Clinic between 2017 and 2024 and diagnosed with SD according to the 2016 American College of Rheumatology/European League Against Rheumatism Classification Criteria [10]. Patients with secondary Sjögren’s syndrome, acute and subacute thyroiditis, toxic adenoma, toxic multinodular goitre, hypothyroidism following thyroid surgery, thyroid dysfunction due to iodine deficiency, subclinical hypothyroidism without the need for medical treatment, hyperthyroidism, thyroid malignancy, and patients with minor salivary gland biopsy results that were suspicious or incompatible with SD were excluded from the study. All diabetic patients included in the study had type 2 diabetes mellitus. The study was approved by the Ethics Committee for Clinical Research (approval number 79/5).

A retrospective evaluation was conducted on the demographic data, biochemical and serological tests, and pathological data of the patients. The Schirmer test and minor salivary gland biopsy were conducted on all patients. The anti-nuclear antibody (ANA) test was performed using the indirect immunofluorescence (IIF) method using HEp-2 (HEp-2000) cells as substrate. The serum sample was stored at +2–8 °C for 3–14 days. The screening dilution was used as 1/160. Values below 1/160 titre were evaluated as negative, 1/160–1/320 1+, 1/320/–1/640 2+, 1/640–1/1280 3+, and >1/1280 4+. In microscopic examination, firstly, positive and negative samples were evaluated at 10× or 20× magnification. Then, positive samples were examined in terms of fluorescent staining pattern (nuclear, cytoplasmic, and mitotic patterns) at 40–60× magnification. The diagnosis of hyperthyroidism and hypothyroidism was based on biochemical tests. Thyroid-stimulating hormone (TSH) > 4.5 mIU/L was defined as hypothyroidism and <0.4 mIU/L as hyperthyroidism. Following the identification of TSH abnormality, patients were evaluated by the Endocrinology Department of our hospital. A comprehensive diagnostic approach is employed, encompassing the assessment of thyroid autoantibodies and ultrasound imaging in all patients with hypothyroidism. Serum samples from patients with hypothyroidism were analysed for anti-thyroglobulin (anti-Tg) and anti-thyroperoxidase (anti-TPO) antibodies, and thyroid ultrasound and fine-needle biopsy were performed on patients with thyroid nodules. Conversely, patients diagnosed with hyperthyroidism underwent a comprehensive diagnostic evaluation that included TSH receptor antibody testing, thyroid ultrasound, and thyroid scintigraphy. In cases where cold nodules were identified on scintigraphy, a thyroid biopsy was performed to obtain a definitive diagnosis. In the study group, 11 patients were diagnosed with hyperthyroidism, while a further 9 patients were diagnosed with thyroid malignancy following a thyroid fine-needle biopsy. An analysis was conducted of the autoantibodies present in patients with malignancy, in order to ascertain any differences when compared with salivary biopsy results. However, due to the limited number of patients, no significant results could be obtained. It is for this reason that these patients were excluded from the study.

The measurement of the rheumatoid factor (RF) test was conducted through the utilisation of the immunoturbidimetric method, while the anti-TG and anti-TPO tests were measured by means of the electrochemiluminescence immunoassay (ECLIA) method.

### Statistical Analysis

The data obtained from the study were subjected to statistical analysis using the SPSS 29.0 (Statistical Package for Social Sciences, IBM, Armonk, NY, USA) software package. Descriptive statistics were used to present categorical data as frequency (percentage ratio) and proportional scale data as mean ± SD. The Kolmogorov–Smirnov method, the student’s *t*-test, and Chi-square analysis were used. The chi-square test was employed for the purpose of analysing the distribution of discrete variables between the groups. In the case of continuous variables, the *t*-test was employed for data that exhibited a normal distribution, whereas the Mann–Whitney U test was utilised for data that did not. All analyses were conducted with a significance level of *p* < 0.05.

## 3. Results

AT was detected in 167 (31.8%) of 525 patients who participated in the study. The mean age of the patients with SD+AT was 60.1 ± 10.9 years. The female sex ratio was significantly higher in the SD+AT group compared to the pSS group (96.4% vs. 88.5%, *p* = 0.003). Upon analysis of the ANA positivity rates, it was observed that the SD+AT group exhibited a statistically significant elevation in ANA positivity compared to the SD group. The anti-Sjögren’s syndrome type A (anti-SS-A) positivity rate was higher in the AT group (*p* = 0.002). Histological examination of salivary gland biopsies revealed a significantly higher prevalence of diffuse lymphocytic infiltration in the SD+AT group compared to the SD group. Lymphoma was found to be significantly higher in the SD+AT group (two patients, 1.1%) compared to the SD group (three patients, 0.8%). However, no statistically significant difference was observed between the two groups. The positivity rates for the Schirmer test were comparable between the two groups and did not achieve statistical significance (83.2% vs. 88%). When the two groups were evaluated in terms of SD pulmonary involvement, it was found that both groups exhibited similar rates of involvement. Concomitantly, the rates of RF positivity were similar between the two groups. Comorbid diseases including hypertension, diabetes mellitus, coronary artery disease, and asthma were similar in both groups (Table 1).

**Figure 1:**
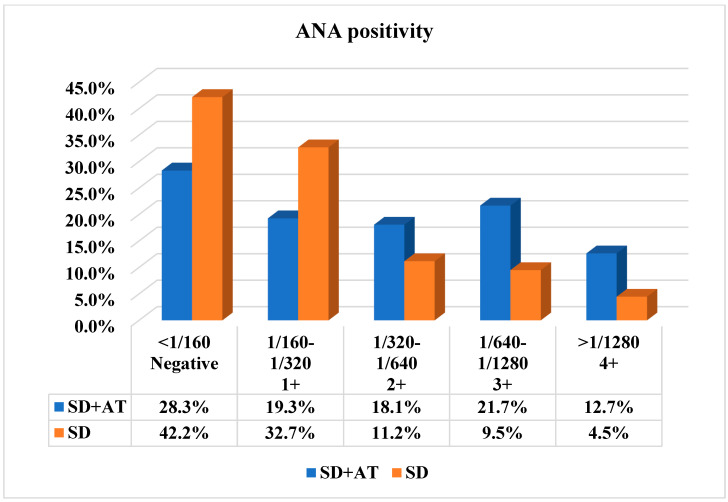
ANA positivity rates and titres. *p* value was <0.01 in all columns. SD: Sjogren Disease, ANA: Anti-nuclear Antibody, AT: Autoimmune thyroiditis.

**Figure 2:**
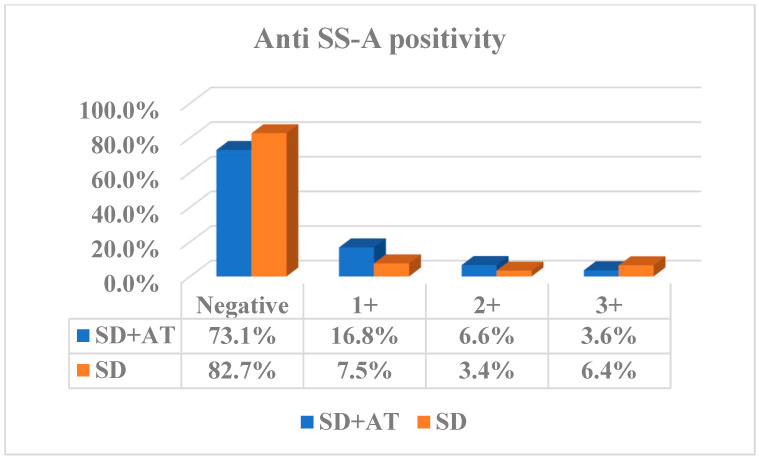
Anti SS-A positivity rates and titres. *p* value was 0.002. SD: Sjogren disease, AT: autoimmune thyroiditis, anti-SS-A: anti-Sjögren’s syndrome type A.

The ANA positivity rate was 71.7% in the SD+AT group and 57.8% in the SD group. The observed difference between the two groups was statistically significant (<0.001). The ANA positivity titres were also found to be significantly higher in the SD+AT group. The ANA positivity titres of 2+ (1/320–1/640), 3+ (1/640–1/1280) and 4+ (>1/1280) were found to be significantly elevated in the AT group (*p* < 0.01) (Figure 1). The anti-SS-A positivity rate was 26.9% in the SD+AT group and 17.3% in the SD group. The difference between the two groups was statistically significant (*p* = 0.002) (Table 1). Anti-SS-A positivity titres were found to be higher in the SD+AT group, especially at 1+ and 2+ titres. No notable discrepancy was identified between the two groups with regard to anti-SS-A 3+ (Figure 2).

In the binary logistic regression analysis of patients, ANA positivity titres were found to be significant at 2+, 3+, and 4+ values. Odd ratios or Exp(B) were found to be 2.41, 3.40, and 4.21, respectively. We found that the likelihood of developing AT increased as ANA titres increased. While the probability of AT is not detected when the ANA is 1+, we observe that the probability of AT increases significantly at 2+, 3+, and 4+ titres (Table 2).

## 4. Discussion

Autoimmune diseases are defined as diseases that cause tissue damage as a result of immune dysregulation. They affect approximately 10% of the global population. It is of great importance to consider the impact of various factors, including gender, socioeconomic status, regional location, and seasonal variations, on the distribution of diseases. In particular, the correlations between rheumatological and endocrinological disorders suggest the involvement of a shared etiological factor and pathological mechanism [4,11]. Our study revealed that AT was present in 31% of patients with SD, a figure that is significantly higher than that observed in the general population. The heightened pathological manifestations and serological indicators observed in this patient group, when compared to patients with SD alone, indicate the potential involvement of analogous immunological pathways in both diseases.

ANA positivity is a valuable biomarker for the diagnosis of SD. In the 2012 ACR SD classification criteria, a titre of 1/320 was accepted as a criterion for diagnosis in cases of ANA positivity. In the recently updated classification criteria, the ANA test was excluded from the criteria set. This is due to the fact that anti-SSA/B(Ro/La) positivity is of greater significance in the diagnosis, with only a small number of cases exhibiting anti-SSA/B(Ro/La) negativity and ANA (titre ≥ 1:320) positivity [10,12]. In clinical practice, the presence of ANA is a significant factor in the selection of patients for minor salivary gland biopsy and in the confirmation of the diagnosis. The prevalence of ANA positivity in the diagnosis of SD is reported to be between 50 and 70%. However, the frequency of this finding varies across studies. In a study by Santiago et al., 218 patients diagnosed with SD were examined. The ANA positivity rate was 62%, and a significant and independent association with minor salivary gland biopsy was identified [13,14]. In the most recently published British Society for Rheumatology guideline, it is emphasised that ANA is widely used as a screening antibody in clinical practice. It is further suggested that ANA can be employed as a screening antibody in cases where there is a clinical suspicion of connective tissue disease [15]. In our study, the ANA positivity rate was found to be 62.2%, which is similar to the rates observed in other studies. This rate was significantly higher in the group with AT compared to the group without AT. Titres above 1/320, which are considered significant, were observed to be higher in the AT group in our study, and the probability of AT increased with increasing titre.

Anti-SS-A positivity is a more specific antibody for SD. A number of studies have demonstrated an association between anti-SS-A positivity and a range of complications, including lymphadenopathy, lymphoma, cytopenia, hypergammaglobulinaemia, and vasculitis. Concurrently, it is established that clinical manifestations, activity indices, and the risk of progression to systemic extraglandular disease are elevated in proportion to the degree of anti-SS-A positivity [16,17]. Similarly, the anti SS-A positivity rates were found to be higher in the AT group. Significant rates were observed, particularly in the 2+ and 3+ titres, where the number of patients was high. This suggests that chronic thyroiditis, which is an organ-specific immune condition, is also associated with systemic immunity, although it is known that it is limited to the organ and does not cause a systemic immune response. On the other hand, with minor salivary gland pathologies, which are one of the most important parameters for the diagnosis of SD, a similar picture is encountered. The prevalence of diffuse lymphocytic infiltration in SD+AT patients is once again elevated, paralleling the frequency of ANA positivity. The data indicate that the thyroid, lacrimal, and salivary glands, which are functionally and structurally similar, are exposed to a comparable lymphocytic infiltration. This suggests that the relationship between the two diseases may be more significant than previously thought. However, a study by Anaya et al. demonstrated that the prevalence of ANA positivity and minor salivary gland biopsy positivity was similar in the SD+AT group and the SD group. It is important to note that the number of patients in the SD+AT group was limited in this study. The author posits that the coexistence of these two common diseases can be better understood as polyautoimmunity rather than as a single disease or a secondary disease [18]. Polyautoimmunity is defined as the presence of more than one autoimmune disease in one person. The coexistence of multiple autoimmune diseases, encompassing both systemic and organ-specific manifestations, has been conceptualised as polyautoimmunity in several studies. The underlying mechanism is believed to be attributable to shared genetic backgrounds and exposure to analogous environmental risk factors. The association of SD with systemic diseases, including rheumatoid arthritis, systemic lupus erythematosus, myositis, and organ-specific diseases such as AT, autoimmune hepatitis, primary biliary cirrhosis, immune thrombocytopenia, and pernicious anaemia, suggests that SD may have an important polyautoimmunity feature. However, it is notable that AT is by far more prevalent in SD patients compared to other organ-specific autoimmune diseases. The increased frequency of lymphoma in Hashimoto thyroiditis is another indication that they share similar pathophysiological pathways [19,20]. In a further study of SD+AT, it was observed that the rates of ANA positivity, anti-SS-A positivity, and extraglandular involvement were similar. This study emphasised the high correlation between SD and AT [21]. Some studies have indicated that genetic predisposition plays a greater role in this association than serological positivity. Various gene variations associated with ATs have been defined, particularly in the HLA region (HLA-DPB1, HLA-DQA1, HLA-DRB1, and HLA-DQB1) [19,22].

It has become increasingly evident in recent years that patients with SD are at an elevated risk of developing organ-specific diseases, including but not limited to Addison disease, coeliac disease, AT, multiple sclerosis, primary biliary cholangitis, and vitiligo. Accordingly, it has been recommended that these conditions be considered with particular care prior to the onset of clinical findings in patients with SD [4,23]. In the course of our study, it was observed that thyroid tests were conducted in the majority of patients with SD, and further investigations were performed in patients with thyroid dysfunction. In a study conducted by Girón-Pïllado et al. with 223 SD patients, 40% of patients with thyroid dysfunction were identified. Additionally, it was observed that at least one-third of the patients were not screened for thyroid disease [24].

Although this retrospective study has some limitations, it offers valuable insights that can inform clinical practice. The data set lacked information pertaining to thyroid ultrasound and thyroid biopsy results for patients with AT. This study could have been strengthened by comparing the serological findings and minor salivary gland pathology with thyroid ultrasound and biopsy findings and determining the relationship between them. Another limitation is that the relationship of SD with other organ-specific diseases could not be determined. Further research involving a larger patient series in a prospective design supported by genetic data, including seronegative patients, may help to clarify the relationship between these diseases and the timing of their onset.

## 5. Conclusions

The prevalence of AT disease is markedly elevated in the SD. To think that these two diseases, which are common in the community, come together coincidentally is to ignore the common pathogenetic mechanisms in both diseases. In our study, the presence of ANA positivity, anti-SS-A positivity, and diffuse lymphocytic infiltration appears to influence the association between these two diseases. In particular, in patients with high-titre serological positivity, autoimmune thyroid diseases should be considered as a potential diagnosis before the onset of clinical findings. It is important to screen for thyroid diseases, especially in patients with high-titre ANA positivity, anti-SS-A positivity, and diffuse lymphocytic infiltration in minor salivary gland biopsy and to realise that the coexistence of these two diseases is more common than we thought.

## Figures and Tables

**Table 1:** Sociodemographic, laboratory, and pathological data of the patients.

Variable	SD+AT (*n* = 167)	SD (*n* = 358)	*p*
Age, year	60.1 ± 10.9	59.9 ± 12.5	NS
Female, *n*	161 (96.4%)	317 (88.5%)	0.003 *
ANA positive, *n*	120 (71,7%)	207 (57.8%)	<0.001 *
Anti-SS-A positive, *n*	45 (26.9%)	62 (17.3%)	0.002 *
Anti-SS-B positive, *n*	8 (4.8%)	13 (3.6%)	NS
RF positive, *n*	21 (12.6%)	58 (16.2%)	NS
Schirmer test positive, *n*	139 (83.2%)	315 (88%)	NS
Pathology			0.009 *
I. Focal	50 (29.9%)	147 (41.1%)	
II. Diffuse	117 (70.1%)	211 (58.9%)	
Anti-Tg positive, *n*	73 (43.7%)		
Anti-TPO positive, *n*	81 (48.5%		
Lymphoma, *n*	2 (1.1%)	3 (0.8%)	NS
Pulmonary involvement	5 (3%)	30 (8.4%)	NS
DM, *n*	26 (15.6%)	62 (17.4%)	NS
Hypertension, *n*	47 (28.1%)	98 (27.5%)	NS
CAD, *n*	7 (4.2%)	23 (6.4%)	NS
Asthma, *n*	20 (12%)	34 (9.5%)	NS

*: Significant at 0.05 level, calculated as mean ± SD. NS: not significant. SD: Sjogren disease, ANA: anti-nuclear antibody, anti-SS-A: anti-Sjögren’s syndrome type A, anti-SS-B: anti-Sjögren’s syndrome type B, AT: autoimmune thyroiditis, RF: rheumatoid factor, anti-Tg: anti-thyroglobulin antibodies, anti-TPO: anti-thyroperoxidase antibody, DM: diabetes mellitus, CAD: coronary artery disease.

**Table 2:** Binary logistic regression analysis of SD+AT patients.

ANA	B	S.E.	Sig.	Exp(B)
1/160–1/320 (1+)	0.129	0.260	0.61	0.82
1/320–1/640 (2+)	0.879	0.294	0.003 *	2.41
1/640–1/1280 (3+)	1.224	0.292	<0.001 *	3.40
>1/1280 (4+)	1.439	0.372	<0.001 *	4.21

*: Significant at 0.05 level. SD+AT: Sjogren disease+ autoimmune thyroiditis, ANA: anti-nuclear antibody, B: unstandardized beta, S.E: Standard Error, Sig.: significant, Exp(B): exponential value of B.

## Data Availability

The data presented in this study are available upon request from the corresponding author, due to restrictions regarding privacy and ethics.

## Abbreviations

The following abbreviations are used in this manuscript:

SD	Sjögren disease
AT	Autoimmune thyroiditis
ANA	Anti-nuclear antibody
IIF	Indirect immunofluorescence
